# Sycosis Non-Parasitica

**Published:** 1886-12

**Authors:** A. R. Davidson

**Affiliations:** Prof. Medical Chemistry and Dermatology, Niagara University, one of the Physicians to the Buffalo Hospital of the Sisters of Charity


### *SYCOSIS NON-PARASITICA.*

By A. R. DAVIDSON, M. D.,

Prof. Medical Chemistry and Dermatology, Niagara University, one of the Physicians to the Buffalo Hospital of the Sisters of Charity.

Several cases of this disease have lately presented themselves to me, having a history and appearance which would indicate that the nature and appropriate treatment of this affection of the skin is not always recognized by the general practitioner. The patients usually present themselves with the part diseased partially hidden under a thick growth of hair, and when this is the case we are never surprised to learn that they have been under treatment for a long time, but have rather grown worse than better. Of the many definitions of the disease that given by Duhring is the simplest and most complete, and I give it in his own words: "Sycosis non-parasitica is a chronic, inflammatory, non-contagious disease, involving the hair follicles, characterized by pustules, papules and tubercles perforated by hairs, accompanied usually with burning sensations."

The disease may appear on any part of the body provided with relatively long hair, but its more frequent seat is the region of the chin and cheek, and particularly the upper lip. Its frequent location on the chin has given rise to the synonym, acne mentagra. English dermatologists frequently use the name acne sycosis, but the appelation acne is a misnomer, as it should be confined to those affections in which the sebaceous glands are chiefly affected. It is, of course, true that in the case of the long hairs the sebaceous glands are really appendages of the hair follicle, but it is to affections of the large and complex glandular structure, to which the lanugo or rudimentary hairs seem accessory, the orifice of their ducts opening directly upon the cutaneous surface, that we would confine the term. The name sycosis is derived from the conical, discrete, reddish papules, visible at the part where the hair makes its exit from the duct of the follicle, and which (by a stretch of imagination) suggests the appearance of the surface of a fig. These papules are painful, giving rise to a burning sensation, occasionally itching, when being picked or torn by the fingers, the pus concretes into a crust at the bottom of the hair. There is marked redness of the surrounding skin, and, more or less, inflammatory thickening also usually exists. The disease extends gradually from the initial lesions, and in chronic cases an important clinical feature is the symmetrical and general involvement of the entire surface. The appearance of one cheek is the counterpart of that of the other. The hairs are usually firmly fixed in these follicles, and depilation is painful, but from those where active suppuration exists they may be removed with ease. The disease gives rise to marked deformity, and the patient, in order to cover it as much as possible, and also from the pain and difficulty in shaving, allows his beard or moustache to grow. As long as he does so he will not recover, indeed, this is a disease which does not get well if left alone. What the exciting cause is, is not easily discovered. All classes of society yield cases; it occurs usually between the ages of twenty-five and fifty. It is, as its name indicates, non-parasitic. It is not contagious. Fox says, it occurs generally in patients markedly debilitated, and especially dyspeptic. Duhring says, it attacks the well-nourished as well as those surrounded by poverty. I have myself met with it in all social conditions, but in the majority of cases the patients have been strong, healthy men. The cause of the peculiar obstinacy is, I think, more easily accounted for. The disease is, in its nature, a simple perifollicular inflammation. In health the motions of the free shaft of the hairs do not irritate the follicle in which it is set. In condition of disease it is quite different, as Hyde says: "Each free hair operates like a lever upon the inflamed ring of tissue which encircles it, whenever by the touch of the hand, by the action of brushing, by currents of air, or by any agency whatever a motion is imparted to it. Every such movement must tease, to a variable degree, the surface beneath, already irritated, and when estimate is made of the hundreds of such movements to which each hair is subjected during a period of twenty-four hours, the relative importance of this apparently insignificant factor may be appreciated."

In the diagnosis of sycosis non-parasitic, we have to distinguish it from tinea sycosis, eczema and a syphilide.

The parasitic and non-parasitic forms of the disease differ in their clinical features as follows (Hyde, fol. 343,): The non-parasitic form is recognizable by—

1. The greater redness of the involved surface.

2. The extension of the disease in advanced cases to larger areas of symmetrical development.

3. The more superficial character of the lesion.

4. The firm implantation of the hair in the follicles and their relative freedom, in all cases, from fractures and relics in the form of stumps.

The parasitic form is peculiar in that it is—

1. Comparatively infrequent.

2. Decidedly less redness of the surface attacked.

3. Its frequent limitation to a circumscribed area.

4. The peculiar lumpy, tubercular, nodular and uneven character of the patch upon which Duhring has laid emphasis; and, lastly,

5. The early loosening of the hairs in their follicles, as also of the occurrence of fractured hair and stumps, exhibiting, usually at the bulb, unmistakable evidence of the nature of the disease and the presence of the tricophyton.

To pustular eczema sycosis often bears a striking likeness, from which, however, it may be known by the absence of oozing, as well as of itching. Eczema, moreover, attacking the beard, would be apt to show itself upon other portions of the face. One of the most useful of any single characteristic is, however, the fact, that in sycosis *each pustule is penetrated, through its center, by a hair.*

From acuminated-pustular syphiloderm, there ought to be no difficulty in distinguishing sycosis. In the syphilide there will always be pustules upon other regions of the face, as well as upon the body. Again, the pustular syphiloderm is much less chronic in its course, and, when long persistent in one locality, is characterized by ulceration and the production of very characteristic crusts.

In several cases which have come under my observation during the last year, the diagnosis of the physician had been "Eczema Barbæ." In each case treatment had been vigorous and long continued and worse than useless, but this result was not the consequence of the error in diagnosis, but chiefly to the neglect of what I consider essential to recovery, either in sycosis or in eczema of the beard, namely—the removal of the hair. To this direction the patient almost invariably demurs; he says the pain of shaving would be unbearable, and, further, without the protection of the beard, the deformity would be so great as to necessitate his staying in the house. A statement of the reason why shaving is absolutely necessary, and the assurance that after the first shaving the pain will be slight, and improvements so rapid that the disfigurement will not be long endured is sufficient for most patients.

The pain of the first shaving is greatly relieved by clipping the hair short and keeping it thoroughly saturated with pure olive oil for about twelve hours, after which a sharp razor will remove the hair without much discomfort, even when there is much tenderness, swelling and pustulation of the skin.

The shaving should be repeated on every second day. The appearance, after the first shaving, fully realizes the patient's anticipation. The red infiltrated skin, further marred by pustules, papules and pustulo-papules, makes him anxiously hope that your prediction of speedy improvement may be fulfilled.

The application of very hot water, in which a little borax is dissolved, the opening of any purulent collection, and this, followed by the application of the following ointment, will, in a few days, produce such an improvement that your patient is satisfied to continue the treatment:

| | | |
|---|---|---|
| ℞ | Ung. Diachylon (Hebra), - - | ℥ iss. |
| | Zinci Oxidi, - - - - | ʒ ii. |
| | Hydr. Ammon. Chlorid., - - | gr. xx. |

Bismuth Sub-Nitratis, - - - ʒ ii.

Ung. Petrolat, - - - q. s. ℥ iii.

M. Ft. Ungt.

This should be spread thickly on cloth and bound upon the affected part. The subsequent treatment is largely that of eczema of equal grade of severity.

If, as sometimes happens, some patches are pustulated, the entire affected spot must be epilated.

Occasionally, too, we meet with cases of sycosis confined to the upper lip, caused by a nasal catarrh. Here the first step is to relieve the catarrh and put a stop to the irritating discharge, and then to remove all the hair from the affected area. In any case of chronic circumscribed sycosis it is better to pull out all hairs seated in mature pustules. They are easily extracted, and it gives free exit to the pus. You thus prevent the entire destruction of the follicle and consequent alopecia.

